# The complete mitochondrial genome of *Neomyia gavisa* (Walker, 1859) (Diptera: Muscidae)

**DOI:** 10.1080/23802359.2026.2742598

**Published:** 2026-10-10

**Authors:** Linwen Zhou, Shuangmei Ding, Ding Yang, Xiaoyan Liu

**Affiliations:** a Hubei Insect Resources Utilization and Sustainable Pest Management Key Laboratory, College of Plant Science and Technology, Huazhong Agricultural University, Wuhan, China; b The Institute of Scientific and Technical Research on Archives, National Archives Administration of China, Beijing, China; c College of Plant Protection, China Agricultural University, Beijing, China

**Keywords:** Muscidae, *Neomyia gavisa*, mitogenome

## Abstract

This study reports the first complete mitochondrial genome sequence of *Neomyia gavisa.* The mitochondrial genome is 16,205 bp in length, with an A + T content of 78.5%. The genome contains 13 protein-coding genes (PCGs), 22 tRNA genes, 2 rRNA genes, and a control region. All PCGs start with ATN except *cox1* (TCG).  Bayesian inference analyses indicate that *Neomyia* is closely related to *Eudasyphora*, and that the tribe Stomoxyini is nested within Muscini. The present mitogenome expands taxon sampling within Muscidae and provides a valuable molecular resource for the reconstruction of evolutionary histories at genus level.

## Introduction

The family Muscidae, commonly known as houseflies, is globally distributed and comprises more than 6,000 species in 225 genera (Bánk et al. 2025). *Neomyia* is a genus within the subfamily Muscinae (Pont 2024) and is characterized by suprasquamal ridge setulose, vein M strongly curved forward toward vein R_4 + 5_ before the apex, and body with metallic blue, purple, or green coloration, which makes them look very much like blowflies (Couri et al. 2006; Fan 2008). The larvae of *Neomyia* are coprophagous and contribute to dung decomposition, whereas adults are anthophilous and may also feed on human and animal excrement, highlighting their ecological significance (Savage and Vockeroth 2010). *Neomyia* is distributed throughout the Old World and Australasia and comprises more than 70 species. Among them, *Neomyia gavisa* (Walker, 1859) is widely distributed in the northern part of the Oriental region and has been recorded from 22 provinces in China (Fan 2008; Vikhrev and Erofeeva 2025).

Owing to the maternal inheritance and relatively conserved structure, mitochondrial genomes have become powerful tools for phylogenetic reconstruction and integrative taxonomy (Li et al. 2023; Park et al. 2025; Semenchenko et al. 2025). However, mitochondrial genomic data for *Neomyia* remain scarce, with only the complete mitochondrial genome of *Neomyia cornicina* reported to date (Pi et al. 2021). In this study, we present the first complete mitochondrial genome sequence of *N. gavisa*, which enhances our understanding of mitochondrial genomics in *Neomyia* and informs the evolutionary relationships within the subfamily Muscinae.

## Materials and methods

Adult male specimens of *Neomyia gavisa* were collected on 18 July 2023 from Xiaocaoba, Yiliang, Zhaotong, Yunnan, China (27.63°N, 104.05°E). Specimens were identified using faunal keys (Xue and Chao 1998; Shinonaga and Thinh 1999). Following morphological identification, the specimens were preserved in absolute ethanol and stored at −20 °C at the Hubei Insect Resources Utilization and Sustainable Pest Management Key Laboratory, Huazhong Agricultural University, Wuhan, China, and assigned the voucher number HZAU0010026 (contact Xiaoyan Liu, liuxiaoyan@mail.hzau.edu.cn).

One individual from the collected samples was selected for sequencing. Genomic DNA was extracted from thorax muscle tissue using a TIANamp Genomic DNA Kit (TIANGEN Co., Ltd., China) and sequenced by Novogene Co., Ltd. (Beijing, China) using paired-end sequencing (PE150) on an Illumina NovaSeq platform. Raw reads were quality-filtered with Fastp (Chen et al. 2018), and *de novo* assembly was performed with IDBA_UD (Peng et al. 2012). Sequencing depth and coverage were calculated in Geneious Prime, and the complete mitochondrial genome was confirmed by examining overlapping homologous regions at both ends and aligning the raw reads. The average sequencing coverage depth for the mitochondrial genome was 490.79×, with a maximum of 1045× and a minimum of 51× (Figure S1). The mitochondrial genome was annotated using the MITOS Web Server (http://mitos2.bioinf.uni-leipzig.de/index.py) and Geneious Prime (Kearse et al. 2012), followed by manual curation. Gene lengths, nucleotide composition, and AT- skew and GC-skew (AT-skew = [A − T]/[A + T] and GC-skew = [G − C]/[G + C] (Perna and Kocher 1995)) were calculated using PhyloSuite v1.2.3 (Zhang et al. 2020; Xiang et al. 2023).

To reconstruct the phylogenetic relationships within Muscinae, 13 mitochondrial genomes representing eight genera were analyzed, comprising 12 published GenBank sequences and one newly sequenced genome, with four species as outgroups (Table S1). PCGs and tRNA/rRNA genes were aligned with MAFFT v7.0 using the E-INS-i and G-INS-i strategies, respectively (Katoh and Standley 2013), trimmed with Gblocks (Talavera and Castresana 2007), and concatenated using Concatenate Sequence. Partitioning by gene and selection of the best scheme and evolutionary models were performed with PartitionFinder 2 v2.1.1 (Table S2) (Lanfear et al. 2017) under the corrected Akaike information criterion (AICc). Bayesian inference was conducted in MrBayes 3.2.7 (Ronquist et al. 2012) with two simultaneous MCMC runs for 3 million generations, sampling every 1,000 generations and discarding the initial 25% as burn-in. Convergence was assessed by monitoring the ASDSF, which decreased below 0.01 (Huelsenbeck et al. 2001; Ronquist et al. 2012).

## Results

### Genome structure analysis

The complete mitochondrial genome of *Neomyia gavisa* (GenBank accession: No. PX393501) is 16,205 bp in length and encodes the typical set of 37 genes (13 protein-coding genes (PCGs), 22 tRNAs, 2 rRNAs, and a control region), with 23 genes on the J-strand and 14 genes on the N-strand (Figure 1). The genome exhibits a pronounced A + T bias (78.5%), a weakly positive AT skew (0.034), and a strongly negative GC skew (−0.195) (Table S3). .

**Figure 1.**
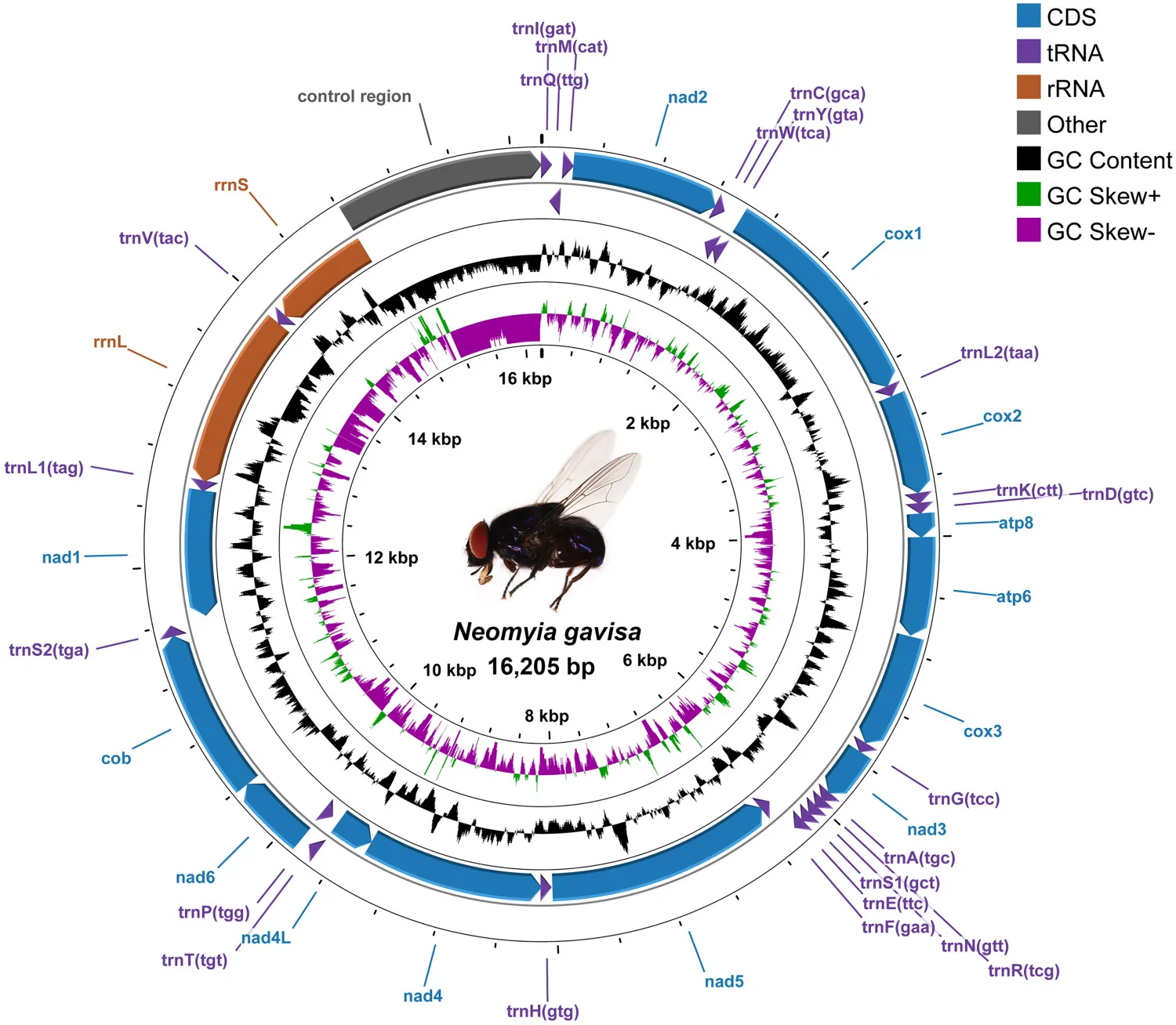
Mitogenome structure of *Neomyia gavisa*. Genes transcribed in the clockwise direction are encoded on the J-strand, transcribed counterclockwise are encoded on the N-strand. The black peaks represent the GC content of the mitogenome, the green peaks represent the positive GC skew, and the purple peaks represent the negative GC skew. Photoed by Linwen Zhou.

Among the 13 PCGs, start codon usage is as follows: ATG start codon in six genes (*cox2*, *cox3*, *nad1*, *nad4*, *nad4L*, and *cytb*), ATT in *nad2*, *nad5* and *nad6*, ATC in *atp8* and *nad3*, ATA in *atp6*, and a unique start codon in *cox1* (TCG). Stop codons comprise six complete TAA (*nad2*, *atp8*, *atp6*, *cox3*, *nad4L*, and *nad6*), three complete TAG (*nad3*, *cytb*, and *nad1*), and four incomplete stop codons consisting of a single T (*cox1*, *cox2*, *nad5*, and *nad4*). The 22 tRNA genes range from 63 to 72 bp. The *rrnS* and *rrnL* genes are 784 bp and 1,328 bp long, respectively, and the control region is 1394 bp. The mitochondrial genome contains 12 intergenic spacer regions and 11 overlapping regions (Table S4).

### Phylogenetic analysis

Phylogenetic analysis based on the mitogenomic dataset (13 PCGs, 22 tRNAs, and two rRNAs; 14,672 bp) robustly supported the monophyly of Muscinae. The tribe Stomoxyini is nested within the tribe Muscini, concordant with prior molecular and morphological evidence that the tribal boundaries within Muscinae may require revision. Within this work, *N. gavisa* and *N. cornicina* form a tight clade together with the genera *Eudasyphora*, highlighting a close evolutionary relationship among these genera (Figure 2).

**Figure 2.**
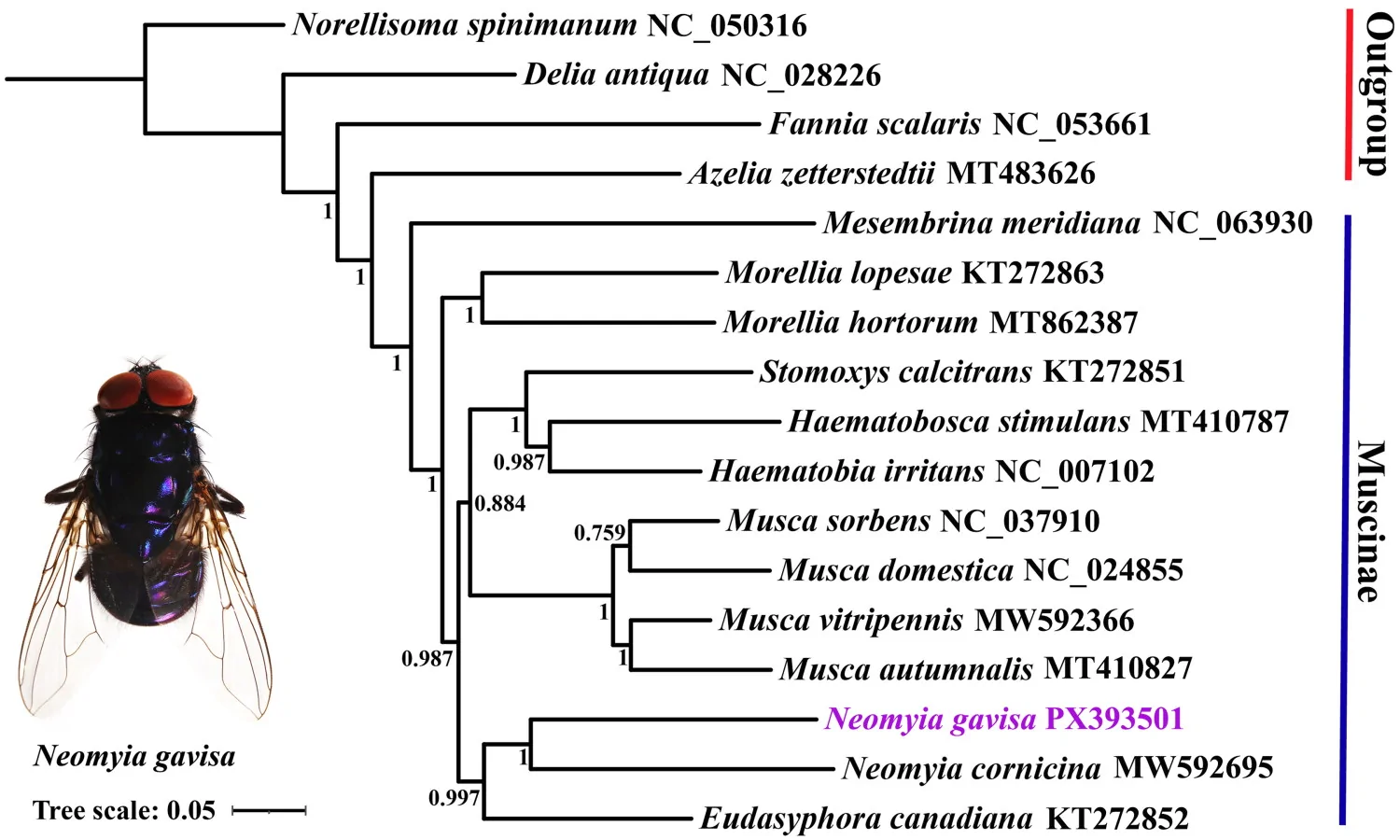
Phylogenetic tree of 17 diptera species (13 species within muscinae) using bayesian inference (BI) based on the 13 shared protein-coding genes, twenty-two tRNAs and two rRNAs. The following sequences were used: *Azelia zetterstedtii* MT483626 (Direct submission), *Delia antiqua* NC_028226 (Zhang et al. 2015), *Eudasyphora canadiana* KT272852 (Junqueira et al. 2016), *Fannia scalaris* NC_053661 (Ren et al. 2020), *Haematobia irritans* NC_007102 (Direct submission), *Haematobosca stimulans* MT410787 (Direct submission), *Mesembrina meridiana* NC_063930 (Direct submission), *Musca autumnalis* MT410827 (Direct submission), *Musca domestica* NC_024855 (Li et al. 2016), *Musca sorbens* NC_037910 (Ma and Huang 2018), *Musca vitripennis* MW592366 (Direct submission), *Morellia hortorum* MT862387 (Direct submission), *Morellia lopesae* KT272863 (Junqueira et al. 2016), *Neomyia cornicina* MW592695 (Pi et al. 2021), *Neomyia gavisa* PX393501 (this study), *Norellisoma spinimanum* NC_050316 (Direct submission), *Stomoxys calcitrans* KT272851 (Junqueira et al. 2016). Photoed by Linwen Zhou.

## Discussion and conclusion

We present the first complete mitochondrial genome sequence of *N. gavisa*. It exhibits a tightly packed gene organization, and its gene order is highly conserved and consistent with the typical mitochondrial gene arrangement in Diptera (Ren et al. 2019; Li et al. 2020).

We analyzed 13 mitochondrial genomes from the subfamily Muscinae to infer phylogenetic relationships and to test the monophyly of Muscinae using a mitogenomic dataset. Bayesian analyses robustly supported the monophyly of Muscinae, consistent with previous studies (Kutty et al. 2014; Haseyama et al. 2015; Grzywacz et al. 2021). However, the taxon sampling in this study is relatively limited, and only mitochondrial genome data were used for phylogenetic analysis. Future studies should expand the taxon sampling and incorporate nuclear gene sequences to further validate and refine the phylogenetic relationships.

This study provides genomic resources for the accurate identification of *N. gavisa*, offers a foundation for future research, confirms existing Muscidae relationships, and reinforces the utility of mitochondrial genomes for resolving tribe-level relationships.

## Supplementary Material

supplementary_material_clean.docx

## Data Availability

The genome sequence data that support the finding of this study is openly available in NCBI (https://www.ncbi.nih.gov), the accession number is PX393501. The associated BioProject, BioSample and SRA numbers are PRJNA1396773, SAMN54383126, and SRR36654926 respectively.

## References

[CIT0001] Bánk O et al. 2025. Catalogue of Life version. 10 Octbor 2025. Catalogue of Life. 10.48580/dgtpl

[CIT0002] Chen S, Zhou Y, Chen Y, Gu J. 2018. fastp: an ultra-fast all-in-one FAST-Q preprocessor. Bioinformatics. 34(17):i884–i890. 10.1093/bioinformatics/bty560 PMC6129281 30423086

[CIT0003] Couri MS, Pont AC, Penny ND. 2006. Muscidae (Diptera) from Madagascar: identification keys, descriptions of new species, and new records. Proc Calif Acad Sci. 57(29):799–923.

[CIT0004] Fan Z. 2008. Fauna Sinica-Insecta. Vol. 49. Diptera Muscidae (1). Science Press p 1–1186.

[CIT0005] Grzywacz A et al. 2021. Towards a new classification of Muscidae (Diptera): a comparison of hypotheses based on multiple molecular phylogenetic approaches. Syst Entomol. 46(3):508–525. 10.1111/syen.12473

[CIT0006] Haseyama KLF, Wiegmann BM, Almeida EAB, de Carvalho CJB. 2015. Say goodbye to tribes in the new house fly classification: a new molecular phylogenetic analysis and an updated biogeographical narrative for the Muscidae (Diptera). Mol Phylogenet Evol. 89:1–12. 10.1016/j.ympev.2015.04.006 25869937

[CIT0007] Huelsenbeck JP, Ronquist F, Nielsen R, Bollback JP. 2001. Bayesian inference of phylogeny and its impact on evolutionary biology. Science. 294(5550):2310–2314. 10.1126/science.1065889 11743192

[CIT0008] Junqueira AC et al. 2016. Large-scale mitogenomics enables insights into Schizophora (Diptera) radiation and population diversity. Sci Rep. 6:21762. 10.1038/srep21762 PMC4766414 26912394

[CIT0009] Katoh K, Standley DM. 2013. MAFFT multiple sequence alignment software version 7: improvements in performance and usability. Mol Biol Evol. 30(4):772–780. 10.1093/molbev/mst010 PMC3603318 23329690

[CIT0010] Kearse M et al. 2012. Geneious Basic: an integrated and extendable desktop software platform for the organization and analysis of sequence data. Bioinformatics. 28(12):1647–1649. 10.1093/bioinformatics/bts199 PMC3371832 22543367

[CIT0011] Kutty SN, Pont AC, Meier R, Pape T. 2014. Complete tribal sampling reveals basal split in Muscidae (Diptera), confirms saprophagy as ancestral feeding mode, and reveals an evolutionary correlation between instar numbers and carnivory. Mol Phylogenet Evol. 78:349–364. 10.1016/j.ympev.2014.05.027 24910153

[CIT0012] Lanfear R, Frandsen PB, Wright AM, Senfeld T, Calcott B. 2017. PartitionFinder 2: new methods for selecting partitioned models of evolution for molecular and morphological phylogenetic analyses. Mol Biol Evol. 34(3):772–773. 10.1093/molbev/msw260 28013191

[CIT0013] Li X et al. 2023. Phylogeny and evolutionary timescale of muscidae (Diptera: Calyptratae) inferred from mitochondrial genomes. Insects. 14(3):286. 10.3390/insects14030286 PMC10059281 36975971

[CIT0014] Li X, Wang Y, Su S, Yang D. 2016. The complete mitochondrial genomes of *Musca domestica* and Scathophaga stercoraria (Diptera: Muscoidea: Muscidae and Scathophagidae). Mitochondrial DNA A DNA Mapp Seq Anal. 27(2):1435–1436. 10.3109/19401736.2014.953080 25163032

[CIT0015] Li XY, Yan LP, Pape T, Gao YY, Zhang D. 2020. Evolutionary insights into bot flies (Insecta: Diptera: Oestridae) from comparative analysis of the mitochondrial genomes. Int J Biol Macromol. 149:371–380. 10.1016/j.ijbiomac.2020.01.249 31991213

[CIT0016] Ma T, Huang J. 2018. The complete mitochondrial genome of the bazaar fly, *Musca sorbens* Wiedemann (Diptera: muscidae). Mitochondrial DNA B Resour. 3(1):436–437. 10.1080/23802359.2018.1450677 PMC7800234 33474195

[CIT0017] Park M, Pyo J, Lee S, Park JS, Kim I. 2025. Complete mitochondrial genome of *Tropilaelaps mercedesae* (Acari: laelapidae): genomic comparison and phylogenetic inference in the dermanyssoidea superfamily. Entomol Res. 55(8):e70057. 10.1111/1748-5967.70057

[CIT0018] Peng Y, Leung HCM, Yiu SM, Chin FYL. 2012. IDBA-UD: a de novo assembler for single-cell and metagenomic sequencing data with highly uneven depth. Bioinformatics. 28(11):1420–1428. 10.1093/bioinformatics/bts174 22495754

[CIT0019] Perna NT, Kocher TD. 1995. Patterns of nucleotide composition at fourfold degenerate sites of animal mitochondrial genomes. J Mol Evol. 41(3):353–358. 10.1007/BF00186547 7563121

[CIT0020] Pi Z, Jiang YS, Wang SJ, Cai JF, Guo YD. 2021. The complete mitochondrial genome of *Neomyia cornicina* (Diptera: muscidae). Mitochondrial DNA B Resour. 6(9):2691–2692. 10.1080/23802359.2021.1966330 PMC8382010 34435121

[CIT0021] Pont AC. 2024. World Muscidae Catalogue. Manuscript, last updated: 07.2024

[CIT0022] Ren L et al. 2020. Comparative analysis of mitochondrial genomes among the subfamily Sarcophaginae (Diptera: sarcophagidae) and phylogenetic implications. Int J Biol Macromol. 161:214–222. 10.1016/j.ijbiomac.2020.06.043 32526299

[CIT0023] Ren LP et al. 2019. Comparative analysis of mitochondrial genomes among four species of muscid flies (Diptera: muscidae) and its phylogenetic implications. Int J Biol Macromol. 127:357–364. 10.1016/j.ijbiomac.2019.01.063 30658142

[CIT0024] Ronquist F et al. 2012. MrBayes 3.2: efficient Bayesian phylogenetic inference and model choice across a large model space. Syst Biol. 61(3):539–542. 10.1093/sysbio/sys029 PMC3329765 22357727

[CIT0025] Savage J, Vockeroth JR 2010. Muscidae (house flies, stable flies). In Brown BV et al. (Eds.), Manual of Central American Diptera. NRC Research Press. Vol 2, p 1281–1295.

[CIT0026] Semenchenko AA, Krasheninnikov AB, Seliverstov NA, Khamenkova EV, Vinnikov KA. 2025. How reliable is mitochondrial DNA for recovering the phylogeny of Chironomidae (Culicomorpha: diptera)? J Insect Sci. 25(4):1. 10.1093/jisesa/ieaf064 PMC12260381 40662267

[CIT0027] Shinonaga S, Thinh TH. 1999. Muscidae of Vietnam 1. Muscinae. Jpn J Syst Ent. 5(2):273–289.

[CIT0028] Talavera G, Castresana J. 2007. Improvement of phylogenies after removing divergent and ambiguously aligned blocks from protein sequence alignments. Syst Biol. 56(4):564–577. 10.1080/10635150701472164 17654362

[CIT0029] Vikhrev NE, Erofeeva EA. 2025. Review of non-afrotropical *Neomyia* Walker, 1859 (Diptera, Muscidae). Tr Mord gos prir zapov im P.G. Smidoviča (Proceedings of the Mordovia State Nature Reserve). 37: p 79–102. 10.24412/cl-31646-2686-7117-2025-37-79-102

[CIT0030] Xiang CY et al. 2023. Using PhyloSuite for molecular phylogeny and tree‐based analyses. iMeta. 2(1):e87. 10.1002/imt2.87 PMC10989932 38868339

[CIT0031] Xue WQ, Chao JM. 1998. Flies of China. Shenyang: Liaoning Science and Technology Publishing House.

[CIT0032] Zhang D et al. 2020. PhyloSuite: an integrated and scalable desktop platform for streamlined molecular sequence data management and evolutionary phylogenetics studies. Mol Ecol Resour. 20(1):348–355. 10.1111/1755-0998.13096 31599058

[CIT0033] Zhang NX et al. 2015. The Complete Mitochondrial Genome of Delia antiqua and Its Implications in Dipteran Phylogenetics. PLoS One. 10(10):e0139736. 10.1371/journal.pone.0139736 PMC4591329 26427045

