# Supplementary material for: The complete mitochondrial genome of Neomyia gavisa (Walker, 1859) (Diptera: Muscidae)
Source: Mitochondrial DNA B Resour. 2026 Oct 10;11(11):1312–6. doi: 10.1080/23802359.2026.2742598 (PMC13656655; doi:10.1080/23802359.2026.2742598)
Supplement: Supplementary file 1 [file TMDN_A_2742598_SM7514.docx]

**Supplementary materials**

Figure S1. Sequencing depth and coverage map of mitochondrial genome of *Neomyia gavisa*.

The final 16,205 bp assembly exhibits uniformly high coverage across the genome, with an average depth of 490.79×, a maximum depth of 1045x, and a minimum depth of 51x.

Table S1 Molecular phylogenetic sample information

| Species | Accession Number | Reference |
| --- | --- | --- |
| *Azelia zetterstedtii* | MT483626 | - |
| *Delia antiqua* | NC_028226 | Zhang *et al*. 2015 |
| *Eudasyphora canadiana* | KT272852 | Junqueira *et al*. 2016 |
| *Fannia scalaris* | NC_053661 | Ren *et al*. 2020 |
| *Haematobia irritans* | NC_007102 | - |
| *Haematobosca stimulans* | MT410787 | - |
| *Mesembrina meridiana* | NC_063930 | - |
| *Musca autumnalis* | MT410827 | - |
| *Musca domestica* | NC_024855 | Li *et al*. 2016 |
| *Musca sorbens* | NC_037910 | Ma and Huang 2018 |
| *Musca vitripennis* | MW592366 | - |
| *Morellia hortorum* | MT862387 | - |
| *Morellia lopesae* | KT272863 | Junqueira *et al*. 2016 |
| *Neomyia cornicina* | MW592695 | Pi *et al*. 2021 |
| *Neomyia gavisa* | PX393501 | this study |
| *Norellisoma spinimanum* | NC_050316 | - |
| *Stomoxys calcitrans* | KT272851 | Junqueira *et al*. 2016 |

Table S2 The partitioning scheme and base substitution models

| Partition | Subset partitions | Best model |
| --- | --- | --- |
| Partition 1 | atp6, cox3 | GTR+I+G |
| Partition 2 | atp8 | TIM+I+G |
| Partition 3 | cox1, cox2 | GTR+I+G |
| Partition 4 | nad3, cytb | GTR+I+G |
| Partition 5 | nad1 | GTR+I+G |
| Partition 6 | nad2 | TIM+I+G |
| Partition 7 | trnF, nad4L | K81UF+I+G |
| Partition 8 | nad4, trnH, nad5 | TIM+I+G |
| Partition 9 | nad6 | GTR+I+G |
| Partition 10 | trnS1, trnQ, trnL1, trnI、trnV, trnY, trnA | TVM+I+G |
| Partition 11 | trnL2, trnW, trnM | TRN+G |
| Partition 12 | trnT, trnS2 | TIM+I+G |
| Partition 13 | trnK, trnR | TVM+I |
| Partition 14 | trnP | K81UF+G |
| Partition 15 | trnN, trnD | GTR+G |
| Partition 16 | rrnL, trnG, rrnS, trnC | GTR+I+G |
| Partition 17 | trnE | F81+G |

Table S3 Nucleotide composition of the mitochondrial genome of *Neomyia gavisa*

| Feature | Length(bp) | T (%) | C (%) | A (%) | G (%) | A+T (%) | G+C (%) | Skew | |
| --- | --- | --- | --- | --- | --- | --- | --- | --- | --- |
|  |  |  |  |  |  |  |  | AT-skew | GC-skew |
| Full genome | 16205 | 37.9 | 12.8 | 40.6 | 8.6 | 78.5 | 21.4 | 0.034 | -0.195 |
| atp6 | 675 | 41.6 | 15.6 | 33 | 9.8 | 74.6 | 25.4 | -0.115 | -0.228 |
| atp8 | 165 | 41.8 | 12.7 | 40.6 | 4.8 | 82.4 | 17.5 | -0.015 | -0.448 |
| cox1 | 1534 | 39 | 14.8 | 31.3 | 14.9 | 70.3 | 29.7 | -0.109 | 0.004 |
| cox2 | 685 | 40 | 13.6 | 34.6 | 11.8 | 74.6 | 25.4 | -0.072 | -0.069 |
| cox3 | 789 | 39.3 | 16.2 | 30.8 | 13.7 | 70.1 | 29.9 | -0.121 | -0.085 |
| cytb | 1137 | 40.5 | 14.6 | 32.7 | 12.2 | 73.2 | 26.8 | -0.106 | -0.089 |
| nad1 | 948 | 49.4 | 8.1 | 28.7 | 13.8 | 78.1 | 21.9 | -0.265 | 0.26 |
| nad2 | 1011 | 44.3 | 11.2 | 36.9 | 7.6 | 81.2 | 18.8 | -0.091 | -0.189 |
| nad3 | 354 | 47.5 | 12.4 | 31.9 | 8.2 | 79.4 | 20.6 | -0.196 | -0.205 |
| nad4 | 1339 | 48.4 | 7.6 | 30.7 | 13.3 | 79.1 | 20.9 | -0.224 | 0.271 |
| nad4L | 291 | 50.2 | 5.5 | 32.3 | 12 | 82.5 | 17.5 | -0.217 | 0.373 |
| nad5 | 1714 | 47.5 | 8.4 | 31.9 | 12.3 | 79.4 | 20.7 | -0.197 | 0.186 |
| nad6 | 525 | 45.5 | 11.2 | 38.3 | 5 | 83.8 | 16.2 | -0.086 | -0.388 |
| PCGs | 11163 | 44.1 | 11.6 | 32.5 | 11.8 | 76.6 | 23.4 | -0.151 | 0.008 |
| tRNA | 1467 | 38.6 | 9.7 | 39.1 | 12.5 | 77.7 | 22.2 | 0.007 | 0.125 |
| rrnL | 1328 | 43 | 5.9 | 39.8 | 11.3 | 82.8 | 17.2 | -0.039 | 0.31 |
| rrnS | 784 | 39.9 | 7.7 | 39.2 | 13.3 | 79.1 | 21 | -0.01 | 0.268 |
| control region | 1394 | 39.7 | 9.3 | 49.8 | 1.2 | 89.5 | 10.5 | 0.112 | -0.767 |

Table S4 Mitogenome structure of *Neomyia gavisa*

| Gene | strand | Location | | Length | Intergenic/ | Codon | |
| --- | --- | --- | --- | --- | --- | --- | --- |
|  |  | Start | Stop | （bp） | Overlap | Start | Stop |
| trnI（gat） | J | 1 | 66 | 66 |  |  |  |
| trnQ（ttg） | N | 64 | 132 | 69 | -3 |  |  |
| trnM（cat） | J | 147 | 215 | 69 | 14 |  |  |
| nad2 | J | 216 | 1226 | 1011 |  | ATT | TAA |
| trnW（tca） | J | 1225 | 1292 | 68 | -2 |  |  |
| trnC（gca） | N | 1285 | 1348 | 64 | -8 |  |  |
| trnY（gta） | N | 1352 | 1417 | 66 | 3 |  |  |
| cox1 | J | 1416 | 2949 | 1534 | -2 | TCG | T |
| trnL2（taa） | J | 2950 | 3015 | 66 |  |  |  |
| cox2 | J | 3020 | 3704 | 685 | 4 | ATG | T |
| trnK（ctt） | J | 3705 | 3775 | 71 |  |  |  |
| trnD（gtc） | J | 3775 | 3842 | 68 | -1 |  |  |
| atp8 | J | 3843 | 4007 | 165 |  | ATC | TAA |
| atp6 | J | 4004 | 4678 | 675 | -4 | ATA | TAA |
| cox3 | J | 4685 | 5473 | 789 | 6 | ATG | TAA |
| trnG（tcc） | J | 5478 | 5542 | 65 | 4 |  |  |
| nad3 | J | 5543 | 5896 | 354 |  | ATC | TAG |
| trnA（tgc） | J | 5895 | 5959 | 65 | -2 |  |  |
| trnR（tcg） | J | 5959 | 6021 | 63 | -1 |  |  |
| trnN（gtt） | J | 6023 | 6087 | 65 | 1 |  |  |
| trnS1（gct） | J | 6088 | 6155 | 68 |  |  |  |
| trnE（ttc） | J | 6156 | 6222 | 67 |  |  |  |
| trnF（gaa） | N | 6243 | 6309 | 67 | 20 |  |  |
| nad5 | N | 6310 | 8023 | 1714 |  | ATT | T |
| trnH（gtg） | N | 8039 | 8103 | 65 | 15 |  |  |
| nad4 | N | 8104 | 9442 | 1339 |  | ATG | T |
| nad4L | N | 9442 | 9732 | 291 | -1 | ATG | TAA |
| trnT（tgt） | J | 9735 | 9799 | 65 | 2 |  |  |
| trnP（tgg） | N | 9800 | 9865 | 66 |  |  |  |
| nad6 | J | 9868 | 10392 | 525 | 2 | ATT | TAA |
| cytb | J | 10392 | 11528 | 1137 | -1 | ATG | TAG |
| trnS2（tga） | J | 11527 | 11593 | 67 | -2 |  |  |
| nad1 | N | 11614 | 12561 | 948 | 20 | ATG | TAG |
| trnL1（tag） | N | 12563 | 12627 | 65 | 1 |  |  |
| rrnL | N | 12628 | 13955 | 1328 |  |  |  |
| trnV（tac） | N | 13956 | 14027 | 72 |  |  |  |
| rrnS | N | 14028 | 14811 | 784 |  |  |  |
| control region | J | 14812 | 16205 | 1394 |  |  |  |
